# Elevated p-Cresyl Sulfate Levels Are Associated with Impaired Endothelial Function in Patients Undergoing Long-Term Peritoneal Dialysis

**DOI:** 10.3390/life16050787

**Published:** 2026-05-08

**Authors:** I-Min Su, Bang-Gee Hsu, Chin-Hung Liu, Ho-Hsiang Chang, Ming-Chun Chen

**Affiliations:** 1 Department of Anesthesiology, Dalin Tzu Chi Hospital, Buddhist Tzu Chi Medical Foundation, Chiayi 62247, Taiwan; 2 School of Medicine, Tzu Chi University, Hualien 97004, Taiwan; 3 Institute of Medical Sciences, Tzu Chi University, Hualien 97004, Taiwan; 4 Division of Nephrology, Hualien Tzu Chi Hospital, Buddhist Tzu Chi Medical Foundation, Hualien 97004, Taiwan; 5 Graduate Institute of Clinical Pharmacy, School of Medicine, Tzu Chi University, Hualien 97004, Taiwan; 6 School of Pharmacy, Tzu Chi University, Hualien 97004, Taiwan; 7 Department of Pediatrics, Hualien Tzu Chi Hospital, Buddhist Tzu Chi Medical Foundation, Hualien 97004, Taiwan

**Keywords:** p-Cresyl sulfate, vascular reactivity index, endothelial function, peritoneal dialysis

## Abstract

p-Cresyl sulfate (PCS) is associated with endothelial injury and adverse cardiovascular outcomes. However, its association with endothelial function in patients on peritoneal dialysis (PD) remains unclear. In this cross-sectional study, 82 patients receiving PD were enrolled. Serum PCS concentrations were quantified using high-performance liquid chromatography–mass spectrometry. Endothelial function was evaluated by digital thermal monitoring (DTM), and vascular reactivity was stratified based on vascular reactivity index (VRI) values into good (>2.0), intermediate (1.0–1.9), and poor (<1.0). Overall, 46.3%, 43.9%, and 9.8% of participants had poor, intermediate, and good vascular reactivity, respectively. Poor reactivity was associated with a higher prevalence of diabetes (*p* = 0.018), old age (*p* < 0.001), higher waist circumference (*p* = 0.013), serum C-reactive protein levels (*p* = 0.010), and PCS levels (*p* < 0.001) and lower diastolic blood pressure (*p* = 0.032) and serum creatinine levels (*p* = 0.005). Higher serum log-transformed PCS levels were associated with reduced VRI after adjustment for covariates (*p* < 0.001). In multivariable models adjusted for potential confounders, PCS was independently associated with poor vascular reactivity (*p* = 0.029), with consistent findings observed across penalized regression analyses (all *p* < 0.001). An inverse relationship was observed between serum PCS levels and endothelial function in patients undergoing PD.

## 1. Introduction

The risk of adverse cardiovascular outcomes and death is markedly elevated among patients undergoing long-term peritoneal dialysis (PD) relative to individuals in the general population. Cardiovascular disease accounts for a substantial proportion of deaths among patients on dialysis and is a major determinant of adverse outcomes in this population [1,2,3]. Additionally, the uremic milieu of advanced kidney disease contributes to vascular injury via the accumulation of retained solutes. As renal function declines, uremic toxins, particularly those poorly removed by conventional dialysis techniques, accumulate in the circulation [4,5]. Among the retained toxins, protein-bound solutes are of particular interest, as their strong bond with albumin limits dialytic clearance, allowing them to persist at biologically active levels in patients with kidney failure [4,6].

Conventional dialysis techniques, including PD, are relatively inefficient at clearing protein-bound solutes compared with small water-soluble molecules [7,8,9]. Thus, several gut-derived metabolites progressively accumulate in patients on long-term dialysis. p-Cresyl sulfate (PCS), a representative protein-bound uremic toxin derived from intestinal microbial metabolism, has been extensively investigated among individuals with chronic kidney disease. Due to its high protein-binding capacity, PCS is poorly eliminated by standard dialysis techniques and tends to accumulate in patients undergoing long-term dialysis [8,10]. Circulating PCS levels reportedly remain elevated despite dialysis treatment and may be involved in the development of various systemic complications in patients with advanced kidney disease [11,12].

Elevated circulating PCS levels have been associated with increased risks of cardiovascular events and mortality [13,14], and experimental evidence suggests that PCS may directly affect the vasculature by promoting atherosclerotic changes and vascular remodeling [15,16]. These findings suggest an association between PCS levels and vascular reactivity impairment in patients with advanced kidney disease. Vascular reactivity represents the ability of blood vessels to appropriately respond to physiological stimuli and is an important component of vascular health. Meanwhile, impaired vascular reactivity is associated with functional limitations and adverse cardiovascular outcomes [17,18]. Moreover, structural and phenotypic alterations of vascular smooth muscle cells, as well as endothelial injury following ischemic or inflammatory insults, may contribute to abnormal vascular responsiveness [19,20]. These findings suggest that vascular reactivity impairment may be a key pathway linking uremic toxins, such as PCS, to cardiovascular complications.

Despite increasing recognition of the vascular toxicity of PCS, its association with functional vascular alterations in patients undergoing PD remains insufficiently characterized. Previous studies have mostly focused on associations between PCS and cardiovascular events or structural vascular changes, with only a few evaluating its potential impact on vascular functional responses in dialysis populations. Assessing vascular reactivity provides important insights into the dynamic regulation of vascular tone and microvascular function, which may precede overt structural vascular disease. Noninvasive approaches, such as digital thermal monitoring (DTM), allow for the assessment of endothelial-dependent vascular responses through the vascular reactivity index (VRI), providing a practical method for evaluating microvascular function clinically. In this study, endothelial function, assessed by VRI, was examined in relation to circulating PCS levels among patients undergoing long-term PD.

## 2. Materials and Methods

### 2.1. Study Population

This cross-sectional study included 82 patients receiving maintenance PD for at least 6 months due to end-stage renal disease at Hualien Tzu Chi Hospital, Taiwan. Participants were recruited between 1 May 2021 and 30 June 2021. This investigation was conducted following approval from the Institutional Review Board at Hualien Tzu Chi Hospital (IRB108-219-A). Ethical standards consistent with the Declaration of Helsinki were followed, and all participants gave written informed consent before taking part in the study. No form of financial compensation was involved.

Participants were excluded if they had an active infection or a history of acute coronary syndrome, cerebrovascular disease, heart failure, malignancy, or amputation. Among the participants, 60 were treated with continuous ambulatory PD (CAPD; Dianeal, Baxter Healthcare, Taipei, Taiwan), undergoing three to five daily exchanges, whereas 22 received automated PD (APD) using a cycling device with four to five nightly exchanges.

Clinical data, including dialysis adequacy parameters, were retrieved from medical documentation. Participants were considered to have diabetes mellitus if they had a fasting plasma glucose level ≥ 126 mg/dL or were receiving antidiabetic treatment. Hypertension was determined according to blood pressure measurements (systolic ≥ 140 mmHg or diastolic ≥ 90 mmHg) or recent use of antihypertensive medications within 2 weeks prior to study entry. Data regarding smoking status and medication use, including angiotensin receptor blockers, β-blockers, calcium channel blockers, fibrates, and statins, were also retrieved from medical records.

### 2.2. Anthropometric Characteristics

Anthropometric data were collected with individuals standing and dressed in light clothing. Body height was determined from the plantar surface to the vertex, whereas body weight was obtained using a calibrated digital scale. Waist circumference was determined at the midpoint between the inferior margin of the ribs and the iliac crest. Measurements were recorded to the nearest 0.5 cm and 0.5 kg for height and weight, respectively. BMI was calculated as weight (kg) divided by height squared (m^2^).

### 2.3. Blood Pressure and Biochemical Measurements

After a minimum overnight fast of 8 h, venous blood samples were collected before the daytime PD exchange. A total of approximately 5 mL of blood was obtained, including a 0.5 mL portion of the sample for complete blood count analysis (Sysmex XS-1000i; Sysmex America, Mundelein, IL, USA). The remaining portion of the sample was processed by centrifugation at 3000× *g* for 10 min to obtain serum for biochemical measurements.

Biochemical parameters, including lipid, metabolic, and renal markers, were measured using an automated system (Siemens Advia 1800; Siemens Healthcare, Erlangen, Germany). Serum iPTH concentrations were measured using a commercial ELISA kit (IBL International, Hamburg, Germany).

Following blood collection, blood pressure was assessed after at least 10 min of rest in the supine posture. Measurements were recorded three times at the right brachial artery using an automated oscillometric device and averaged for analysis.

### 2.4. Determination of Serum PCS Levels Using High-Performance Liquid Chromatography–Mass Spectrometry

Serum PCS concentrations were quantified using high-performance liquid chromatography–mass spectrometry (HPLC–MS) with a Waters e2695 system coupled to an ACQUITY QDa mass detector (Waters Corporation, Milford, MA, USA). PCS was quantified using HPLC–MS under standard analytical conditions, with single-ion monitoring at m/z 187.0. Chromatographic separation was performed on a C18 column under standard gradient conditions using a formic acid–methanol mobile phase.

Quantification was based on peak area comparison with standard calibration curves, all of which showed high linearity (r^2^ > 0.995). Data were processed using Empower^®^ 3.0 software, and PCS was detected at a retention time of approximately 16.56 min.

### 2.5. Assessment of Endothelial Function

DTM with a VENDYS-II system (Endothelix Inc., Houston, TX, USA) was applied to evaluate endothelial function. Participants were instructed to avoid smoking, alcohol consumption, caffeinated beverages, and vasoactive medications prior to the evaluations.

Measurements were conducted under controlled temperature conditions (approximately 24 °C) after participants rested for at least 15 min. Blood cuff monitoring was placed on the right upper arm, with temperature sensors secured to the index fingers of both hands, with the left finger serving as the control. Baseline temperature was recorded for 5 min, followed by rapid cuff inflation to 50 mmHg above the systolic blood pressure for 5 min to induce arterial occlusion. Following cuff deflation, reactive hyperemia was generated, and fingertip temperature changes were continuously recorded.

The VRI was calculated as the maximal temperature rebound above baseline during the hyperemic phase. Based on VRI values ranging from 0.0 to 3.5, vascular reactivity was stratified as poor (<1.0), intermediate (1.0–1.9), or good (≥2.0). All values were automatically calculated using VENDYS-II software (https://www.vendys2.com/).

### 2.6. Statistical Methods and Analysis

All analyses were carried out using SPSS (version 25.0; IBM Corp., Armonk, NY, USA) and R software (version 4.2.2). Normality of continuous variables was evaluated using the Shapiro–Wilk test. Normally distributed data are reported as mean ± standard deviation, whereas variables with non-normal distributions are presented as median and interquartile range. Differences between groups were assessed using one-way analysis of variance or the Kruskal–Wallis test, depending on the data distribution. Categorical data are summarized as frequencies and percentages and compared using the chi-square test. Skewed variables, including PD vintage, fasting glucose, triglycerides, iPTH, CRP, total and urine creatinine clearance, and PCS levels, were subjected to logarithmic transformation before analysis.

Initial associations between clinical variables and VRI were assessed using simple linear regression analysis. Variables that showed statistical significance were subsequently included in a multivariable stepwise linear regression model to identify independent determinants of VRI. For linear regression analyses, β coefficients represent the change in VRI per one-unit increase in the independent variable.

To identify predictors of vascular reactivity dysfunction (intermediate vascular reactivity and poor vascular reactivity) or poor vascular reactivity, multivariable logistic regression analysis was performed. Variables with *p* < 0.05 in univariate analyses were included in the multivariable model to avoid excluding potentially relevant predictors, which is a commonly used strategy in exploratory analyses with limited sample sizes. The variables entered into the model included diabetes, age, waist circumference, diastolic blood pressure, serum total cholesterol, creatinine, CRP, and PCS. To further assess model stability and reduce the risk of overfitting, given the relatively small sample size, penalized logistic regression analyses were conducted using LASSO (least absolute shrinkage and selection operator), Ridge regression, and Elastic Net regularization as supportive approaches. Optimal hyperparameters were determined using 5-fold cross-validation. As conventional *p*-values are not applicable in penalized regression models, statistical inference was conducted using bootstrap resampling with 1000 iterations to estimate 95% confidence intervals and empirical *p*-values for the standardized coefficients. A variable was considered statistically significant if the bootstrap 95% confidence interval did not cross 0 and the empirical *p*-value was <0.05. For logistic regression analyses, β coefficients represent log-odds. In penalized models, standardized coefficients were reported to allow comparison across variables. Correlations between log-transformed serum PCS levels and clinical variables were analyzed using Spearman’s rank correlation. Statistical significance was defined as a two-tailed *p*-value < 0.05. Discriminative performance of serum PCS for vascular reactivity dysfunction and poor vascular reactivity was assessed by receiver operating characteristic (ROC) curve analysis, with the corresponding area under the curve (AUC) reported.

## 3. Results

### 3.1. Clinical Characteristics According to Vascular Reactivity

The baseline clinical characteristics of all participants on PD are shown in Table 1. Based on the VRI, 8 (9.8%), 36 (43.9%), and 38 (46.3%) participants were classified as having good, intermediate, and poor vascular reactivity, respectively.

Individuals with reduced vascular reactivity were older (*p* < 0.001) and exhibited greater waist circumference (*p* = 0.013). In addition, diastolic blood pressure varied significantly across the three groups (*p* = 0.032). Additionally, serum creatinine levels were lower (*p* = 0.005), and CRP levels were significantly higher (*p* = 0.010), in patients with poorer vascular reactivity. The prevalence of diabetes mellitus was also greater in the poor vascular reactivity group (*p* = 0.018).

Notably, serum PCS levels differed markedly among the vascular reactivity groups (*p* < 0.001), with the highest levels observed in patients with poor vascular reactivity.

### 3.2. Determinants of VRI

Initial relationships between clinical factors and VRI were explored using simple linear regression, as shown in Table 2. VRI showed significant negative correlations with age (*r* = −0.442, *p* < 0.001), waist circumference (*r* = −0.355, *p* = 0.001), serum log-transformed CRP (*r* = −0.351, *p* = 0.001), and log-transformed PCS levels (*r* = −0.578, *p* < 0.001). Meanwhile, VRI showed positive correlations with diastolic blood pressure (*r* = 0.229, *p* = 0.039), serum total cholesterol levels (*r* = 0.231, *p* = 0.037), and creatinine levels (*r* = 0.278, *p* = 0.011).

In the multivariable analysis, age (β = −0.245, *p* = 0.008), waist circumference (β = −0.233, *p* = 0.008), and serum log-transformed PCS levels (β = −0.452, *p* < 0.001) remained independently associated with VRI.

### 3.3. Predictors of Vascular Reactivity Dysfunction

Multivariable logistic regression that included age, waist circumference, diastolic blood pressure, serum total cholesterol, creatinine, CRP, and PCS levels was performed to identify predictors of vascular reactivity dysfunction (*p* < 0.05; see Table 1). In this analysis, PCS was not significantly associated with the combined vascular reactivity dysfunction group in the conventional multivariable model; however, Ridge (*p* < 0.001) and Elastic Net (*p* = 0.009) regression showed significant associations between PCS and vascular reactivity dysfunction. The lack of statistical significance observed in the conventional multivariable and LASSO models in Table 3 may, in part, be related to the limited sample size of the reference group (good vascular reactivity, *n* = 8). The small number of events in this category could reduce statistical power and increase the uncertainty of the estimated coefficients, particularly in models that do not incorporate shrinkage or regularization. In contrast, Ridge and Elastic Net models, which are less sensitive to small sample sizes and multicollinearity due to coefficient shrinkage, demonstrated significant associations between PCS and vascular reactivity dysfunction. These findings suggest that the association between PCS and vascular dysfunction remains present but may be attenuated when intermediate and poor VRI categories are combined.

### 3.4. Predictors of Poor Vascular Reactivity

Multivariable logistic regression that included diabetes, age, waist circumference, diastolic blood pressure, serum total cholesterol, creatinine, CRP, and PCS levels was performed to identify predictors of poor vascular reactivity (*p* < 0.05; see Table 1). Results showed that PCS had a significant positive association with poor vascular reactivity (β = 11.23, 95% CI: 1.18–21.29, *p* = 0.029, Table 4).

Penalized logistic regression analyses revealed that PCS remained consistently correlated with poor vascular reactivity across all penalized models (all *p* < 0.001) (Table 3). Meanwhile, CRP showed significance only in the Ridge (*p* = 0.044) and Elastic Net (*p* = 0.048) models. Other variables, including age, waist circumference, diastolic blood pressure, serum creatinine, total cholesterol, and diabetes status, were not significantly associated with poor vascular reactivity.

### 3.5. Correlations Between PCS and Clinical Factors

Spearman correlation analysis was conducted to explore associations between serum log-transformed PCS levels and clinical variables (Table 5). PCS levels were positively correlated with age (*r* = 0.346, *p* = 0.001) and serum log-transformed CRP (*r* = 0.262, *p* = 0.017), whereas serum log-transformed PCS levels were negatively correlated with serum albumin levels (*r* = −0.256, *p* = 0.020), creatinine levels (*r* = −0.335, *p* = 0.002), and VRI (*r* = −0.578, *p* < 0.001). There were no significant associations between PCS levels and blood pressure, lipid profile, glucose levels, dialysis adequacy indices, or mineral metabolism markers.

### 3.6. Discrimination and Calibration Performance of PCS

ROC analysis demonstrated that serum PCS had good discriminative ability for vascular reactivity dysfunction, with an AUC of 0.841 (95% CI: 0.720–0.933, *p* < 0.001). The optimal PCS cutoff value was 7.65 mg/L, yielding a sensitivity of 63.51% and a specificity of 100.0%. Calibration analysis indicated an acceptable model fit, with a Hosmer–Lemeshow *p*-value of 0.504 and a Brier score of 0.072 (Table 6). For poor vascular reactivity, PCS showed excellent discriminative performance, with an AUC of 0.947 (95% CI: 0.840–1.000, *p* < 0.001). The optimal cutoff value was 9.72 mg/L, with a sensitivity of 94.74% and a specificity of 100.0%. Calibration was also acceptable, as reflected by a Hosmer–Lemeshow *p*-value of 0.713 and a Brier score of 0.030. Overall, PCS demonstrated stronger discriminative performance for identifying poor vascular reactivity than for the broader category of vascular reactivity dysfunction, suggesting that PCS may be more closely associated with more severe endothelial impairment.

## 4. Discussion

In this cross-sectional analysis, higher serum PCS levels were associated with lower VRI values, suggesting impaired endothelial function. Patients with poorer vascular reactivity exhibited markedly higher serum PCS levels, which had a strong inverse correlation with VRI values. Both regression analyses confirmed the association between PCS levels and vascular reactivity. This association remained robust after penalized regression analysis. These findings suggest that elevated serum PCS levels are closely linked to vascular reactivity impairment in patients undergoing PD.

Participants with poorer vascular reactivity were older and showed higher serum CRP levels and a higher prevalence of diabetes mellitus. These results are aligned with previous reports that aging, systemic inflammation, and metabolic disorders promote vascular reactivity impairment and impaired endothelial regulation [21,22,23]. Aging is linked to progressive vascular stiffening and reduced endothelial responsiveness, while chronic inflammation may promote vascular injury through oxidative stress and endothelial activation [24]. Despite adjusting for these cardiovascular risk factors, our analyses showed that PCS remained independently associated with vascular reactivity impairment, indicating that the observed association between PCS and endothelial dysfunction is not solely explained by traditional risk factors. An additional observation of this study was that lower serum creatinine levels were observed in patients with poorer vascular reactivity. This observation may initially appear counterintuitive, as higher serum creatinine levels are often associated with more advanced renal dysfunction. However, in patients undergoing PD, serum creatinine levels are strongly influenced by non-renal factors, particularly muscle mass. Lower serum creatinine levels may therefore reflect reduced muscle mass or sarcopenia, which are common in older and frail individuals [25]. In addition, low serum creatinine levels may be indicative of poor nutritional status or protein-energy wasting, both of which have been associated with adverse cardiovascular outcomes [26]. Thus, the association between lower serum creatinine levels and impaired vascular reactivity is more likely to reflect underlying frailty and malnutrition rather than better renal function.

The robust association between PCS and vascular reactivity in this study is biologically plausible. PCS exerts direct adverse effects on vascular endothelial cells. For example, it increases endothelial permeability through Src-dependent phosphorylation of vascular endothelial cadherin [27]. In patients with chronic kidney disease, serum PCS levels are associated with endothelial injury biomarkers, supporting its potential role in endothelial dysfunction [28]. Furthermore, PCS promotes vascular pathology by enhancing atherosclerotic changes and vascular remodeling [15]. A key mechanism underlying these vascular effects may involve oxidative stress. PCS has been shown to increase reactive oxygen species and free radical production in various cellular models [29,30]. PCS may also impair antioxidant defenses by reducing intracellular glutathione levels, thereby rendering vascular cells more susceptible to oxidative injury [31]. Through these mechanisms, PCS may be associated with disturbances in vascular homeostasis and endothelial responsiveness. Clinical studies have shown that higher serum PCS levels are associated with adverse cardiovascular outcomes in patients with chronic kidney disease [14].

The observed association between PCS and vascular reactivity may be interpreted from multiple perspectives. In addition to a potential direct biological role, PCS may also reflect the broader uremic milieu characterized by the accumulation of protein-bound solutes, systemic inflammation, and metabolic disturbances. In this context, elevated serum PCS levels may serve as an integrative marker of these underlying processes that are closely linked to vascular reactivity impairment. This perspective is supported by the observed correlations between PCS, inflammatory markers, and serum albumin levels in the present study. Accordingly, PCS may capture the cumulative burden of uremic toxicity rather than acting as a single isolated mediator of vascular injury. As a gut-derived uremic toxin originating from intestinal microbial metabolism, PCS levels may also be influenced by dietary protein intake and gut microbiota composition, both of which may act as potential confounders in the observed associations. Future longitudinal and mechanistic studies are needed to clarify whether PCS is directly involved in vascular dysfunction or primarily reflects the underlying uremic environment.

The findings of this study may also be interpreted in the context of the limited removal of protein-bound uremic solutes during dialysis. Compared with small water-soluble molecules, these toxins are less efficiently cleared by conventional dialysis modalities, including PD [7,8,9]. Gut-derived metabolites such as PCS can persist in the circulation of patients on long-term dialysis, reflecting the incomplete elimination of protein-bound compounds [8,9,10]. Therefore, the retention of these solutes may represent an important component of the uremic environment in patients on PD. Consequently, serum PCS levels may provide additional information on the metabolic and vascular disturbances observed in individuals on chronic dialysis therapy [4].

The clinical implications of these findings warrant consideration. Vascular reactivity represents dynamic vascular function and may provide critical information beyond structural vascular alterations. Functional vascular abnormalities may precede overt structural vascular disease. They may also contribute to early cardiovascular risk development [18]. Impaired vascular reactivity is also associated with functional limitations and adverse clinical outcomes in patients with vascular disorders [17]. Therefore, the observed association between serum PCS levels and reduced VRI may also provide insights into the vascular health of patients on PD. The consistent association observed across multiple regression approaches further supports the robustness of the relationship between serum PCS levels and vascular reactivity impairment. Importantly, similar associations were observed when VRI was analyzed as a continuous variable and across the original three-category classification, supporting the robustness of the findings. Uremic toxins are an integral component of metabolic disturbances in dialysis populations. Monitoring serum PCS levels may therefore help characterize the uremic milieu and its relationship with vascular reactivity impairment [4]. In contrast, traditional clinical variables such as age and diabetes were not retained as significant predictors in the penalized models. This may be explained by the shared pathophysiological pathways linking these factors to vascular reactivity impairment. Age, diabetes, and systemic inflammation are closely interrelated and may contribute to endothelial impairment through overlapping mechanisms [32]. In this sense, PCS may act as an integrative biomarker reflecting the cumulative burden of these processes. Moreover, penalized regression methods are designed to address multicollinearity by selecting variables with the most stable and independent associations. Therefore, the lack of statistical significance for certain clinical variables does not necessarily indicate a lack of biological relevance.

The definition and categorization of VRI require careful consideration. In the present study, intermediate and poor VRI categories were combined to define vascular reactivity dysfunction based on the conceptual distinction between normal and abnormal vascular function. Both categories represent impaired endothelial responsiveness compared with good VRI and are therefore clinically relevant as indicators of endothelial dysfunction. In addition, this approach improved statistical stability, given the relatively small number of participants with good vascular reactivity. Nevertheless, we acknowledge that this categorization may reduce granularity and introduce clinical heterogeneity, as the intermediate group likely includes patients with milder vascular impairment. To address this limitation, we performed sensitivity analyses using alternative outcome definitions. The association between PCS and vascular reactivity remained consistent when VRI was analyzed as a continuous variable and when poor vascular reactivity was evaluated as a separate outcome. Notably, the association was stronger and more consistent for poor VRI, suggesting that PCS may be more closely related to more severe endothelial dysfunction. These findings highlight the importance of outcome definition in interpreting associations with vascular biomarkers and suggest that PCS may have greater clinical relevance in identifying advanced vascular dysfunction rather than early or intermediate abnormalities.

In addition to outcome definition, the discrimination and calibration analyses further supported this interpretation. In the present study, PCS demonstrated good discriminative ability for vascular reactivity dysfunction and excellent performance for identifying poor vascular reactivity. Notably, the higher AUC observed for poor vascular reactivity suggests that PCS is more strongly associated with more severe endothelial dysfunction rather than with milder or intermediate abnormalities. In addition, calibration metrics indicated acceptable model fit for both outcome definitions, as reflected by non-significant Hosmer–Lemeshow test results and relatively low Brier scores. However, given the limited sample size and the small number of participants in the good VRI group, the calibration results should be interpreted with caution. In summary, these results suggest that PCS may have greater clinical utility in identifying patients with advanced vascular dysfunction rather than serving as a general screening marker for early endothelial impairment. Future studies with larger cohorts are needed to further validate these findings and to assess the predictive performance of PCS in longitudinal settings.

This study has several limitations. First, the cross-sectional design precludes causal inference between serum PCS levels and endothelial dysfunction, and the observed associations should be interpreted accordingly. In addition, the possibility of reverse causation cannot be excluded, as impaired vascular function or underlying disease severity may also influence serum PCS levels. Moreover, the relatively low events-per-variable ratio, owing to the limited number of events (*n* = 38) related to the number of predictors, may have increased the susceptibility to overfitting. This concern is partly addressed by the use of penalized regression techniques to improve model stability. Second, as this investigation was conducted at a single center in Taiwan and involved a modest sample size, the generalizability of the findings may be constrained. However, to mitigate potential overfitting and improve the stability of the estimates, we applied penalized regression techniques and bootstrap resampling. Despite these considerations, the results should be interpreted with caution and further validated in larger, independent cohorts. Moreover, the generalizability of the findings to other populations should be interpreted with caution. Ethnic and dietary differences may influence PCS levels, as gut microbiota composition and dietary protein intake are known to affect the generation of protein-bound uremic toxins. Third, despite incorporating multiple clinical covariates into multivariable analyses, residual confounding related to unmeasured factors, including dietary patterns, gut microbiota composition, and other uremic toxins, cannot be entirely ruled out. Fourth, only total PCS levels were measured in this study, whereas the free (unbound) fraction is considered the biologically active component. As PCS is highly protein-bound, variations in protein binding may influence its bioavailability and toxicity. Therefore, total PCS levels may not fully reflect the biologically active fraction, and the observed associations should be interpreted with this limitation in mind. Finally, endothelial function was evaluated using DTM rather than invasive vascular testing; however, this technique offers a noninvasive and reproducible method for evaluating microvascular reactivity in clinical settings. Future prospective studies with larger cohorts would help to further clarify the temporal relationship between serum PCS levels and vascular reactivity impairment in patients on PD.

The present findings have potential clinical implications. Serum PCS may serve as a risk stratification biomarker for impaired vascular reactivity in patients undergoing PD, particularly for identifying those with more severe endothelial dysfunction. Because PCS is a uremic toxin originating from gut microbial metabolism, future studies should evaluate whether interventions targeting the gut–kidney axis, including strategies targeting diet, the gut microbiota, and toxin adsorption, can reduce PCS levels and improve vascular function. In addition, dialysis innovations aimed at enhancing the removal of protein-bound toxins, including adsorption-based approaches and novel membrane technologies, may represent important future therapeutic directions. Prospective and interventional studies are needed to determine whether reducing PCS can translate into improved vascular and cardiovascular outcomes.

## 5. Conclusions

Overall, elevated serum PCS levels showed an independent association with impaired endothelial function among patients receiving PD. This relationship remained consistent across multiple regression models, indicating a robust association between serum PCS levels and vascular reactivity impairment. These findings highlight the potential relevance of protein-bound uremic toxins in vascular abnormalities observed in this population. Further prospective studies are needed to clarify the temporal relationship and clinical significance of PCS in cardiovascular risk assessment among patients receiving PD.

## Figures and Tables

**Table 1:** Clinical characteristics according to different vascular reactivity indexes by digital thermal monitoring of the 82 peritoneal dialysis patients.

Characteristics	All Patients (*n* = 82)	Good Vascular Reactivity (*n* = 8)	Intermediate Vascular Reactivity (*n* = 36)	Poor Vascular Reactivity (*n* = 38)	*p*-Value
Age (years)	57.17 ± 14.56	42.50 ± 10.53	54.03 ± 15.37	63.24 ± 11.21	<0.001 *
PD vintage (months)	48.54 (20.79–81.51)	54.42 (21.69–76.74)	52.74 (27.00–84.57)	28.74 (18.45–74.79)	0.273
Height (cm)	160.40 ± 8.76	162.88 ± 10.49	160.81 ± 9.50	159.50 ± 7.71	0.578
Body weight (kg)	65.10 ± 14.43	62.99 ± 19.26	64.73 ± 13.51	65.89 ± 14.54	0.860
Body mass index (kg/m^2^)	25.17 ± 4.52	23.37 ± 4.75	24.86 ± 3.58	25.84 ± 5.20	0.326
Waist circumference (cm)	91.07 ± 11.12	82.50 ± 11.64	89.64 ± 9.62	94.24 ± 11.37	0.013 *
Vascular reactivity index	1.04 ± 0.65	2.34 ± 0.18	1.35 ± 0.24	0.47 ± 0.29	<0.001 *
Systolic blood pressure (mmHg)	150.96 ± 22.59	152.38 ± 19.79	147.61 ± 20.36	153.84 ± 25.13	0.492
Diastolic blood pressure (mmHg)	88.44 ± 15.40	101.00 ± 16.00	88.81 ± 14.14	85.45 ± 15.43	0.032 *
Hemoglobin (g/dL)	9.66 ± 1.40	8.93 ± 1.28	9.67 ± 1.46	9.81 ± 1.34	0.269
Total cholesterol (mg/dL)	154.85 ± 48.33	191.75 ± 40.02	153.53 ± 47.45	148.34 ± 48.38	0.066
Triglyceride (mg/dL)	113.50 (77.75–183.75)	80.00 (60.25–161.50)	120.50 (79.00–205.25)	121.50 (81.25–183.00)	0.324
Fasting glucose (mg/dL)	112.50 (94.75–137.00)	98.00 (94.50–116.50)	111.00 (91.25–136.25)	114.50 (97.50–141.25)	0.518
Albumin (g/dL)	3.54 ± 0.35	3.61 ± 0.37	3.60 ± 0.31	3.46 ± 0.38	0.217
Blood urea nitrogen (mg/dL)	60.67 ± 18.21	62.50 ± 14.41	59.67 ± 16.79	61.24 ± 20.44	0.895
Creatinine (mg/dL)	10.57 ± 3.35	13.43 ± 3.66	11.04 ± 3.36	9.53 ± 2.86	0.005 *
Total calcium (mg/dL)	9.63 ± 0.62	9.57 ± 1.02	9.63 ± 0.48	9.64 ± 0.66	0.949
Phosphorus (mg/dL)	5.27 ± 1.35	5.66 ± 1.46	5.47 ± 1.41	4.99 ± 1.24	0.221
iPTH (pg/mL)	178.20 (80.53–452.78)	295.10 (145.85–722.98)	209.05 (85.15–520.43)	161.05 (54.35–360.05)	0.438
C-reactive protein (mg/dL)	0.20 (0.11–0.98)	0.15 (0.10–0.23)	0.14 (0.10–0.54)	0.36 (0.14–1.70)	0.010 *
Total p-Cresyl sulfate (mg/L)	8.67 (2.72–23.06)	1.80 (0.82–6.81)	3.94 (2.15–7.79)	24.19 (14.37–34.28)	<0.001 *
Weekly Kt/V	1.95 ± 0.42	2.20 ± 0.86	1.93 ± 0.36	1.93 ± 0.33	0.229
Peritoneal Kt/V	1.77 ± 0.39	1.97 ± 0.62	1.76 ± 0.39	1.73 ± 0.34	0.291
Total Clcr (L/week)	54.26 (47.67–64.04)	52.11 (42.37–67.73)	56.15 (48.58–68.20)	53.62 (46.70–61.85)	0.740
Peritoneal Clcr (L/week)	47.28 ± 12.44	45.07 ± 12.63	48.24 ± 13.03	46.83 ± 12.08	0.777
Urine Clcr (mL/min)	0.01 (0.00–1.23)	0.55 (0.00–1.66)	0.00 (0.00–1.13)	0.12 (0.00–1.31)	0.814
Female, *n* (%)	48 (58.5)	4 (50.0)	20 (55.6)	24 (63.2)	0.702
Diabetes, *n* (%)	36 (43.9)	2 (25.0)	11 (30.6)	23 (60.5)	0.018 *
Hypertension, *n* (%)	65 (79.3)	6 (75.0)	29 (80.6)	30 (78.9)	0.938
APD, *n* (%)	22 (26.8)	2 (25.0)	7 (19.4)	13 (34.2)	0.356
ARB use, *n* (%)	47 (57.3)	5 (62.5)	23 (63.9)	19 (50.0)	0.460
β-blocker use, *n* (%)	32 (39.0)	3 (37.5)	17 (47.2)	12 (31.6)	0.385
CCB use, *n* (%)	45 (54.9)	3 (37.5)	24 (66.7)	18 (47.4)	0.145
Statin use, *n* (%)	33 (40.2)	5 (62.5)	15 (41.7)	13 (34.2)	0.324
Fibrate use, *n* (%)	18 (22.0)	1 (12.5)	1 (30.6)	6 (15.8)	0.245

Values for continuous variables are given as means ± standard deviation and tested by one-way analysis of variance; variables not normally distributed are given as medians and interquartile range and tested by Kruskal–Wallis analysis; values are presented as numbers (%) and analyzed after analysis by the chi-square test. iPTH, intact parathyroid hormone; APD, ambulatory peritoneal dialysis; Weekly Kt/V, weekly fractional clearance index for urea; Clcr, creatinine clearance; ARB, angiotensin-receptor blocker; CCB, calcium-channel blocker. * *p* < 0.05 was considered statistically significant after Kruskal–Wallis analysis or one-way analysis of variance.

**Table 2:** Correlation of vascular reactivity index levels and clinical variables by simple or multivariable linear regression analyses among 82 peritoneal dialysis patients.

Variables	Vascular Reactivity Index				
	Simple Regression		Multivariable Regression		
	*r*	*p* Value	Beta	Adjusted R^2^ Change	*p*-Value
Age (years)	−0.442	<0.001 *	−0.245	0.046	0.008 *
Log-PD vintage (months)	0.115	0.302	–	–	–
Body mass index (kg/m^2^)	−0.209	0.060	–	–	–
Waist circumference (cm)	−0.355	0.001 *	−0.233	0.060	0.008 *
Systolic blood pressure (mmHg)	−0.087	0.435	–	–	–
Diastolic blood pressure (mmHg)	0.229	0.039 *	–	–	–
Hemoglobin (g/dL)	−0.185	0.095	–	–	–
Total cholesterol (mg/dL)	0.231	0.037 *	–	–	–
Log-Triglyceride (mg/dL)	−0.038	0.736			
Log-Glucose (mg/dL)	0.119	0.285	–	–	–
Albumin (g/dL)	0.160	0.140	–	–	–
Blood urea nitrogen (mg/dL)	−0.039	0.727	–	–	–
Creatinine (mg/dL)	0.278	0.011 *	–	–	–
Total calcium (mg/dL)	0.031	0.784	–	–	–
Phosphorus (mg/dL)	0.069	0.540	–	–	–
Log-iPTH (pg/mL)	0.168	0.131	–	–	–
Log-CRP (mg/dL)	−0.351	0.001 *	–	–	–
Log-PCS (mg/L)	−0.578	<0.001 *	−0.452	0.326	<0.001 *
Weekly Kt/V	0.039	0.728	–	–	–
Peritoneal Kt/V	0.091	0.415	–	–	–
Log-Total Clcr (L/week)	−0.083	0.458	–	–	–
Peritoneal Clcr (L/week)	0.005	0.961	–	–	–
Log-Urine Clcr (mL/min)	0.056	0.730	–	–	–

Data on PD vintage, fasting glucose, triglycerides, iPTH, CRP, total Clcr, urine Clcr, and PCS showed skewed distributions and were log-transformed before analysis. Data analysis was performed using simple linear regression or multivariable stepwise linear regression (adapted factors included age, waist circumference, diastolic blood pressure, serum total cholesterol, creatinine, log-CRP, and log-PCS). Abbreviations as in Table 1. * *p* < 0.05 was considered statistically significant.

**Table 3:** Multivariable and penalized logistic regression analyses of vascular reactivity dysfunction (intermediate or poor), presented as adjusted odds ratios and 95% confidence intervals.

Factors	Multivariable β (95% CI)	Multivariable *p*-Value	LASSO β (95% CI)	LASSO *p*-Value	Ridge β (95% CI)	Ridge *p*-Value	Elastic Net β (95% CI)	Elastic Net *p*-Value
p-Cresyl sulfate (mg/L)	0.175 (−0.067–0.416)	0.157	0.061 (0.000–0.120)	0.072	0.046 (0.021–0.068)	<0.001 *	0.063 (0.007–0.100)	0.009 *
Age (years)	0.012 (−0.070–0.093)	0.777	0.019 (0.000–0.105)	0.529	0.025 (−0.006–0.059)	0.146	0.022 (0.000–0.086)	0.402
Waist circumference (cm)	0.081 (−0.046–0.208)	0.210	0.041 (0.000–0.123)	0.272	0.040 (−0.012–0.082)	0.071	0.046 (0.000–0.109)	0.146
DBP (mmHg)	−0.038 (−0.129–0.054)	0.420	−0.021 (−0.071–0.006)	0.375	−0.023 (−0.054–0.013)	0.184	−0.024 (−0.077–0.011)	0.317
Diabetes, present	−0.392 (−2.851–2.068)	0.755	0.000 (−1.110–0.680)	1.000	−0.147 (−1.174–0.918)	0.783	0.000 (−1.490–1.298)	1.000
Total cholesterol (mg/dL)	−0.023 (−0.048–0.003)	0.083	−0.012 (−0.030–0.000)	0.149	−0.012 (−0.023–−0.002)	0.024 *	−0.013 (−0.026–0.000)	0.070
C-reactive protein (mg/dL)	1.228 (−3.745–6.201)	0.628	0.000 (0.000–0.220)	1.000	0.160 (0.052–0.355)	0.040	0.022 (0.000–0.429)	0.838
Creatinine (mg/dL)	−0.263 (−0.602–0.075)	0.127	−0.142 (−0.424–0.000)	0.245	−0.137 (−0.269–0.005)	0.064	−0.156 (−0.383–0.000)	0.155

Factors associated with vascular reactivity dysfunction were examined using multivariable logistic regression. Covariates demonstrating statistical significance in univariate analyses (Table 1) were included in the model, namely diabetes, age, waist circumference, diastolic blood pressure, serum total cholesterol, creatinine, C-reactive protein, and p-Cresyl sulfate. To enhance model stability and address potential overfitting, particularly in light of the sample size, additional analyses were conducted using penalized regression approaches (LASSO, Ridge, and Elastic Net). Parameter uncertainty was quantified through bootstrap resampling (1000 iterations), from which adjusted odds ratios, 95% confidence intervals, and empirical *p*-values were estimated. LASSO, least absolute shrinkage and selection operator; β, standardized coefficients; CI, confidence interval. * *p* < 0.05 was considered statistically significant (two-sided).

**Table 4:** Multivariable and penalized logistic regression analyses of poor vascular reactivity, presented as adjusted odds ratios and 95% confidence intervals.

Factors	Multivariable β (95% CI)	Multivariable *p*-Value	LASSO β (95% CI)	LASSO *p*-Value	Ridge β (95% CI)	Ridge *p*-Value	Elastic Net β (95% CI)	Elastic Net *p*-Value
p-Cresyl sulfate (mg/L)	11.23 (1.18, 21.29)	0.029 *	6.47 (5.08, 8.91)	<0.001 *	4.16 (3.42, 4.71)	<0.001 *	4.85 (4.05, 5.61)	<0.001 *
Age (years)	0.74 (−1.31, 2.78)	0.480	0.56 (0.00, 1.84)	0.224	0.57 (−0.11, 1.55)	0.136	0.54 (−0.07, 1.56)	0.212
Waist circumference (cm)	0.22 (−2.58, 3.02)	0.878	0.05 (−0.21, 1.25)	0.992	0.11 (−0.62, 0.88)	0.716	0.10 (−0.46, 1.02)	0.768
DBP (mmHg)	0.19 (−1.97, 2.34)	0.864	0.00 (−0.93, 1.09)	0.824	0.26 (−0.39, 1.04)	0.472	0.15 (−0.51, 0.96)	0.684
Diabetes, present	0.90 (−1.39, 3.19)	0.441	0.43 (−0.08, 1.34)	0.580	0.23 (−0.39, 1.09)	0.428	0.27 (−0.32, 1.14)	0.436
Total cholesterol (mg/dL)	−0.44 (−3.39, 2.50)	0.769	−0.36 (−1.59, 0.14)	0.100	−0.31 (−0.87, 0.27)	0.240	−0.30 (−0.95, 0.18)	0.208
C-reactive protein (mg/dL)	1.00 (−1.42, 3.41)	0.419	0.78 (0.00, 1.87)	0.128	0.85 (0.04, 1.49)	0.044 *	0.82 (0.01, 1.53)	0.048 *
Creatinine (mg/dL)	0.25 (−2.14, 2.63)	0.839	−0.11 (−1.24, 0.08)	0.116	−0.37 (−1.10, 0.12)	0.164	−0.29 (−1.14, 0.17)	0.192

Factors associated with vascular reactivity dysfunction were examined using multivariable logistic regression. Covariates identified in univariate analyses (Table 1) were included in the model, namely diabetes, age, waist circumference, diastolic blood pressure, serum total cholesterol, creatinine, C-reactive protein, and p-Cresyl sulfate. To enhance robustness and address potential overfitting, particularly in the context of the sample size, penalized regression approaches were additionally applied. Parameter uncertainty was quantified using bootstrap resampling (1000 iterations), and adjusted odds ratios, 95% confidence intervals, and empirical *p*-values were derived accordingly. LASSO, least absolute shrinkage and selection operator; β, standardized coefficients; CI, confidence interval. * *p* < 0.05 was considered statistically significant (two-sided).

**Table 5:** Associations between log serum p-Cresyl sulfate levels and clinical factors.

Variables	Spearman’s Correlation Coefficient	*p*-Value
Age (years)	0.346	0.001 *
Log-PD vintage (months)	−0.078	0.489
Body mass index (kg/m^2^)	0.090	0.424
Waist circumference (cm)	0.174	0.117
Systolic blood pressure (mmHg)	0.080	0.476
Diastolic blood pressure (mmHg)	−0.176	0.114
Hemoglobin (g/dL)	0.079	0.478
Total cholesterol (mg/dL)	−0.135	0.228
Log-Triglyceride (mg/dL)	−0.006	0.959
Log-Glucose (mg/dL)	0.118	0.289
Albumin (g/dL)	−0.256	0.020 *
Blood urea nitrogen (mg/dL)	−0.054	0.631
Creatinine (mg/dL)	−0.335	0.002 *
Total calcium (mg/dL)	−0.005	0.962
Phosphorus (mg/dL)	−0.030	0.790
Log-iPTH (pg/mL)	−0.054	0.633
Log-CRP (mg/dL)	0.262	0.017 *
Vascular reactivity index	−0.578	<0.001 *
Weekly Kt/V	−0.107	0.340
Peritoneal Kt/V	−0.036	0.747
Log-Total Clcr (L/week)	−0.142	0.204
Peritoneal Clcr (L/week)	−0.004	0.973
Log-Urine Clcr (mL/min)	−0.170	0.287

Data on PD vintage, fasting glucose, triglycerides, iPTH, CRP, total Clcr, urine Clcr, and PCS showed skewed distributions and were log-transformed before analysis. Abbreviations as in Table 1. * *p* < 0.05 was considered statistically significant (2-tailed).

**Table 6:** Performance of PCS in discriminating and calibrating vascular reactivity dysfunction (intermediate or poor) and poor vascular reactivity.

Outcome	Events/Total	AUC (95% CI) *p*-Value	Optimal PCS Cutoff	Sensitivity	Specificity	Brier Score	Hosmer–Lemeshow *p*-Value
Vascular reactivity dysfunction	74/82	0.841 (0.720–0.933) <0.001 *	7.65 mg/L	63.51%	100.0%	0.072	0.504
Poor vascular reactivity	38/82	0.947 (0.840–1.000) <0.001 *	9.72 mg/L	94.74%	100.0%	0.030	0.713

Vascular reactivity dysfunction was defined as intermediate or poor vascular reactivity versus good vascular reactivity. AUC, area under the receiver operating characteristic curve; PCS, p-cresyl sulfate; CI, confidence interval. Calibration was assessed using the Hosmer–Lemeshow goodness-of-fit test and Brier score. * *p* < 0.05 was considered statistically significant.

## Data Availability

Data generated and analyzed in the present study can be obtained from the corresponding author upon reasonable request but are not publicly available owing to ethical and privacy constraints.

## Abbreviations

The following abbreviations are used in this manuscript:

APD	Automated peritoneal dialysis
AUC	Area under the curve
BMI	Body mass index
CAPD	Continuous ambulatory peritoneal dialysis
Clcr	Clearance of creatinine
CRP	C-reactive protein
DTM	Digital thermal monitoring
HPLC–MS	High-performance liquid chromatography–mass spectrometry
iPTH	Intact parathyroid hormone
Kt/V	Fractional clearance index for urea
LASSO	Least absolute shrinkage and selection operator
PD	Peritoneal dialysis
PCS	p-Cresyl sulfate
VRI	Vascular reactivity index
